# Novel combined single dose anti-hepatitis C therapy: a pilot study

**DOI:** 10.1038/s41598-021-84066-3

**Published:** 2021-02-25

**Authors:** Gamal Shiha, Reham Soliman, Mohamed Elbasiony, Noureldien H. E. Darwish, Shaker A. Mousa

**Affiliations:** 1 Internal Medicine Department, Faculty of Medicine, Mansoura University, Mansoura, Egypt; 2 Egyptian Liver Research Institute and Hospital (ELRIAH), Mansoura, Egypt; 3 Tropical Medicine Department, Faculty of Medicine, Port Said University, Port Said, Egypt; 4 Hematology Unit, Clinical Pathology Department, Faculty of Medicine, Mansoura University, Mansoura, Egypt; 5 The Pharmaceutical Research Institute, Albany College of Pharmacy and Health Sciences, 1 Discovery Drive, Rensselaer, NY 12144 USA; 6 Virothera Pharmaceuticals, Rensselaer, NY USA

**Keywords:** Drug development, Hepatitis

## Abstract

The new anti-hepatitis C virus (HCV) molecules improve treatment regimens and outcomes, but there are drawbacks. New combinations should target the HCV infectious cycle and be effective against all HCV genotypes. We developed the novel formulation Catvira, composed of epigallocatechingallate (EGCG) + sofosbuvir + ribavirin. Here, we compared Catvira to sofosbuvir + ribavirin tablets in patients with CHC genotype 4 in a randomized open-label efficacy and safety study. Treatment-naïve and treatment-experienced patients (n = 80) were randomly assigned to receive a single daily fixed dose of Catvira or sofosbuvir + ribavirin for 12 or 24 weeks. Both Catvira and sofosbuvir + ribavirin yielded similar outcomes of viral load (*p* < 0.001). Patients receiving Catvira had a significantly more rapid rate of viral load decline with sustained virologic response (SVR12) achieved by 90% of patients receiving 12 weeks of treatment. Catvira did not impact hemoglobin levels while sofosbuvir + ribavirin showed significant decline in hemoglobin levels after 24 weeks (*p* < 0.05). In this clinical trial (ClinicalTrials.gov Identifier NCT02483156), we found that Catvira administered daily for 12 or 24 weeks is safe, effective, and well-tolerated in both naïve and treatment-experienced patients with HCV genotype 4.

## Introduction

According to the World Health Organization (WHO) report in 2020, 71 million people have chronic hepatitis C virus (HCV) infection globally with annul deaths of 400,000 from hepatitis C-related liver diseases^1^. The prevalence of HCV infection has markedly declined after the availability of direct acting antivirals (DAAs)^2^. Egypt records the highest HCV prevalence worldwide. HCV genotype 4 represents 93% of infections, while HCV genotype 1 never exceeds 10%^2,3^.

Recently, new regimens involving DAA agents have been approved for the treatment of HCV^4–6^. However, there are still drawbacks. The ideal inhibitor should work against more than one HCV target, including viral entry, replication, and assembly/secretion. Also, ribavirin (RBV), which is still commonly used in combination with other drugs for treatment of HCV infection, often induces hemolytic anemia even with a reduction of RBV dose^7^. However, there is still an important role for RBV in HCV treatment, particularly in patients with features that make a cure difficult, i.e., treatment-experienced and cirrhotic^8,9^.

Sofosbuvir (SOF) was the first interferon (IFN)-free DAA to be developed. In addition to its pangenotypic activity, it can be safely combined with other DAA molecules to achieve high sustained virologic response (SVR) rates in almost all HCV genotypes^4^. According to the recently published European Association for the Study of Liver recommendations, the combinations SOF/ledipasvir, SOF/daclatasvir (DCV), or SOF/velpatasvir are all considered optimal options for patients with HCV genotypes 1 and 4^10,11^.

Epigallocatechingallate (EGCG), the most important polyphenol component of green tea, has been found to have antiviral and anticancer activity^12^. Green tea catechins include epigallocatechin-3-gallate (EGCG, which accounts for ~ 50% of the total green tea catechins), epigallocatechin (EGC), epicatechingallate (ECG), and epicatechin (EC). They are structurally different due to the presence or absence of a galloyl moiety and the wide variety of hydroxyl groups on the B-ring. For instance, EC, which lacks the gallic acid ester moiety of EGCG and one additional phenolic hydroxyl group, was inactive against HCV^13^. Recently, EGCG was recognized as an inhibitor of HCV access, and it targeted viral cellular entry into hepatoma cell lines and primary human hepatocytes^14^. Also, EGCG has been reported to enhance HCV double stranded RNA-induced antiviral innate immune responses^13^. Clinical studies have shown that EGCG is safe and well tolerated in healthy human volunteers^13–16^.

In our recently published study (ClinicalTrials.gov Identifier NCT03186313), in patients with CHC genotype 4 we used different formulations of Dactavira (EGCG + SOF + DCV, without RBV) compared with the standard of care treatment (SOF + DCV), and Dactavira plus (EGCG + SOF + DCV + RBV) compared with the standard of care plus RBV^17^. In this current study (ClinicalTrials.gov Identifier NCT02483156), we used the novel formulation of Catvira (EGCG + SOF + RBV) compared with standard of care treatment (SOF + RBV) in a different group of patients with CHC genotype 4.

The aim of this phase 3, open-label study was to compare the novel formulation (EHCV, Catvira), which is composed of EGCG 400 mg + SOF 400 mg + RBV 1000 mg, to SOF 400 mg + RBV 1000 mg individual tablets in patients with CHC genotype 4. Our hypothesis was that this novel formulation effectively inhibits viral entry into human host cells in addition to improving anti-hemolytic effects.

## Methods

### Patients

The study was conducted in accordance with the guidelines of Good Clinical Practice and was approved by the Research Ethics Committee of the Faculty of Medicine, Mansoura University, number PR/54, and is registered at ClinicalTrials.gov Identifier NCT02483156. All patients were provided written informed consent. Patients were screened and enrolled in this pilot study at a single center, Egyptian Liver Research Institute and Hospital (ELRIAH), Egypt, between July, 2015 and October, 2016. Inclusion criteria were the same as in our previous publication^17^. In brief, this study included only DAA-naïve, non-cirrhotic, genotype 4 infected patients with male to female ratio of 1:1. Patients were required to be more than 18 years of age, have body mass index ≥ 18 kg/m^2^ and have chronic genotype 4 HCV infection with a serum HCV RNA level ≥ 104 log IU/ml. Patients with normal liver function tests, renal function tests, hemoglobin level, and platelets > 50,000/µl were included. Patients with any other diseases (e.g., HBV, HIV, and DM) were excluded. Patients could be either treatment-naïve or treatment-experienced; prior treatment with an anti-HCV direct-acting antiviral was exclusionary. Baseline patient characteristics have been included in Table 1.

**Table 1:** Baseline characteristics of patients.

	Median (IQR) or frequency (%)
**Median age** (years)	42.0 (37.0–55.0)
**Gender**	
Males	40 (50%)
Females	40 (50%)
**HCV viral load**	
Log_10_ PCR	6.24 (5.4–6.81)
**Liver function test**	
ALT (U/L)	30.55 (25.24–48.13)
AST (U/L)	28.50 (21.00–39.00)
Total Bilirubin (mg/dL)	0.48 (0.40–0.80)
Albumin (g/dL)	4.90 (4.00–5.1)
AFP (ng/mL)	3.10 (1.95–5.36)
**Hematological evaluation**	
Hemoglobin Level (g/dL)	14.00 (12.80–15.40)
WBCs (/cmm^3^)	5.80 (4.23–8.52)
Platelets Count (/cmm^3^)	240.0 (200.0–290.0)

### Study design

Treatment-naïve and treatment-experienced patients with genotype 4 HCV infection (n = 80) were randomly assigned to receive a single daily fixed dose of Catvira or SOF + RBV individual tablets daily for 12 or 24 weeks. Treatment-naïve was defined as having never received treatment for HCV with any IFN, RBV, or other approved or experimental HCV-specific DAAs. Treatment-experienced was defined as IFN intolerant, non-response, or patient with relapse/breakthrough. The random allocation sequence was generated with computer software (EDGAR, Norwich, England).

It was planned that an approximately even number of treatment-naïve and treatment-experienced subjects would be enrolled across the 2 treatment arms. In Arm 1, subjects received SOF + RBV as one tablet for a total dose of SOF 400 mg orally once daily in the morning and 1000 mg RBV divided into 2 doses daily (600 mg in the morning and 400 mg in the evening) and with food, for 12 or 24 weeks. In Arm 2, subjects received a single dose of Catvira as two tablets once daily with each tablet containing SOF 200 mg, RBV 500 mg, and EGCG 200 mg and with food, for 12 or 24 weeks.

### Efficacy assessments

As in our previous publication^17^, serum HCV RNA was measured using the COBAS TaqMan HCV Test v2.0 (Roche Molecular Systems, Pleasanton, CA, USA). Assessment of the serum HCV RNA was performed at baseline, during treatment, and 4–12 weeks post-treatment. Patients with confirmed virologic relapse that occurred at the end of treatment or in post-treatment visits were ruled out. On-treatment virologic failure was defined as: breakthrough, i.e., confirmed HCV RNA ≥ LLOQ after having previously had HCV RNA < LLOQ while on-treatment; rebound, i.e., confirmed > 1-log^10^ IU/ml increase in HCV RNA from nadir while on-treatment; or, non-response, i.e., HCV RNA persistently ≥ LLOQ through 8 weeks of treatment. Relapse was defined as confirmed HCV RNA ≥ LLOQ during the post-treatment period having achieved HCV RNA < LLOQ at the end of treatment^17^.

### Safety assessments

Safety was evaluated by assessment of clinical laboratory tests, physical examination, vital sign measurements, and documentation of adverse events. Safety data were collected from the first dose of study medication through 30 days after the last dose.

### Statistical assessments

The primary efficacy endpoint was the proportion of all randomized patients who achieved SVR 12 weeks after the end of treatment (SVR12). Secondary efficacy endpoints included SVR4 and SVR24, on-treatment virologic failure, and virologic relapse after the end of treatment. In the primary efficacy analysis, SVR12 rates were calculated for each treatment group, along with 2-sided 95% confidence intervals (CIs) based on the Clopper-Pearson exact method. Differences between groups were considered statistically significant for p ≤ 0.05. All statistical analyses were performed using GraphPad InStat 3 (GraphPad, San Diego, CA, USA).

### Role of the funding source

The ELRIAH had a role in the study design, data collection, and data analysis. The corresponding author had full access to all the data in the study and had final responsibility for the decision to submit for publication.

### Ethics approval

The study (ClinicalTrials.gov Identifier NCT02483156, 26/06/2015) was conducted in accordance with the guidelines of Good Clinical Practice and was approved by the Independent Review Board of the Faculty of Medicine, Mansoura University. All patients provided written informed consent. Special thanks to the ELRIAH clinical trial team and Virothera LLC for supporting the manufacturing and QC for Catvira clinical batches.

## Results

### Patients

Out of 92 screened patients with chronic HCV, 80 met the inclusion criteria, and 40 were enrolled in Arm 1 and completed the treatment with SOF 400 mg + RBV 1000 mg for 12 or 24 weeks. The other 40 patients were enrolled in Arm 2 and completed Catvira treatment with EGCG 400 mg + SOF 400 mg + RBV 1000 mg for 12–24 weeks.

### Efficacy

**Figure 1:**
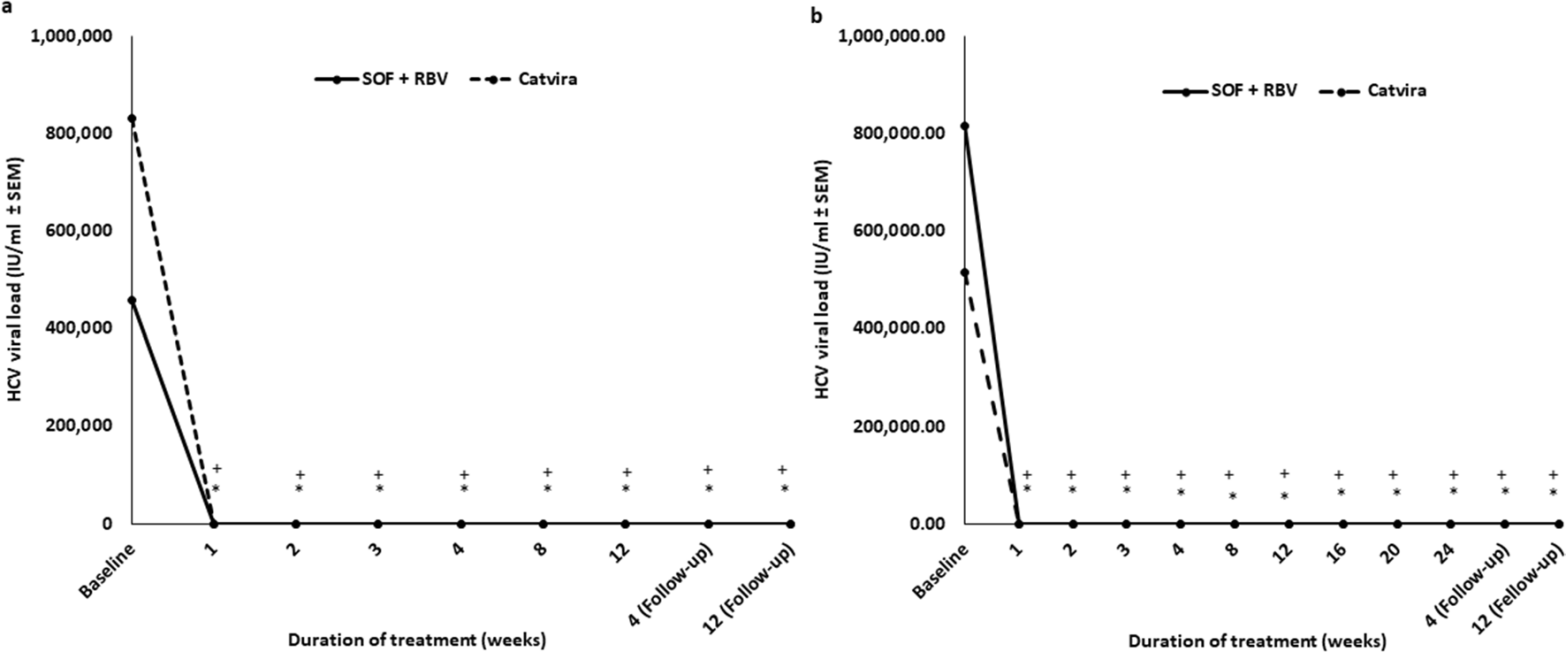
Effects on the viral load (IU/ml) of Catvira treatment versus sofosbuvir (SOF) + ribavirin (RBV) individual tablets treatment in treatment-experienced patients with HCV through (**a**) 12 and (**b**) 24 weeks, n = 80. One-way ANOVA was used followed by the Newman-Keuls post-test, *p < 0.001 represents comparison of both groups SOF + RBV (*) and Catvira (+) versus their baseline. There was a significant trend for rapid lowering of HCV viral load in both groups (SOF + RBV and Catvira) versus their baseline (*p* < 0.001).

There was a significant trend for rapid lowering of HCV viral load in both groups (SOF + RBV and Catvira) versus their baseline (*p* < 0.001). Both SOF + RBV individual tablets and Catvira treatments were effective, with no statistically significant difference regarding SVR12 for both the 12-week (except in week 2) or 24-week study periods. Patients receiving Catvira showed rapid decline in HCV RNA levels upon initiation of treatment, from a mean of 831,425 IU/ml at baseline to 143 IU/ml in the 12-week group and from 515,633.3 IU/ml to 89 IU/ml in the 24-week group after 1 week of treatment. On the other hand, patients receiving SOF + RBV individual tablets showed decline in HCV RNA levels upon initiation of treatment, from a mean of 457,231 IU/ml at baseline to 691 IU/ml in the 12-week group and from 816,392.5 IU/ml to 96 IU/ml in the 24-week group after 1 week of treatment (Fig. 1).

Regarding the Catvira group, SVR12 was achieved by 18 patients (90.0%) receiving 12 weeks of treatment and by 17 patients (85.0%) receiving 24 weeks of treatment. However, for patients receiving SOF + RBV, SVR12 was achieved by 19 patients (95.0%) receiving 12 weeks of treatment and by the 20 patients (100%) receiving 24 weeks of treatment.

### Safety

Adverse effects in the Catvira group were reported in 4 patients (5%) in the 24-week group and were predominantly mild or moderate in severity. The most common adverse events in both groups were headache and epigastric pain (Table 2). In general, Catvira-related adverse effects were similar or even less than in the SOF + RBV treatment group and for a shorter duration. All these adverse events resolved and no severe adverse events were reported.

**Table 2:** Number of adverse events observed and in patients with sofosbuvir (SOF) + ribavirin (RBV) individual tablets and Catvira, n = 80, through 12 and 24 weeks.

	SOF + RBV	Catvira
Headache	5 (6.25%)	2 (2.5%)
Epigastric pain	2 (2.5%)	2 (2.5%)
Insomnia	2 (2.5%)	0
Constipation	1 (1.25%)	0

**Figure 2:**
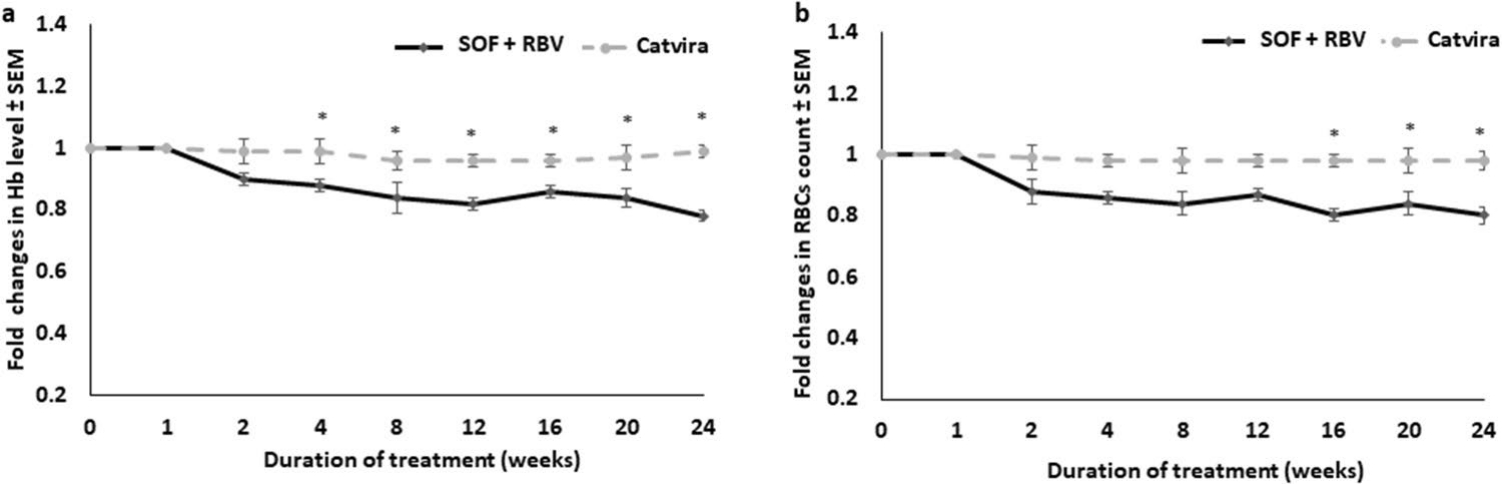
Effects on hematological parameters of Catvira treatment versus sofosbuvir (SOF) + ribavirin (RBV) individual tablets treatment in treatment-experienced patients with HCV (24 weeks treatment). (**a**) Fold changes in hemoglobin levels. (**b**) Fold changes in RBC counts. Values are expressed as mean ± SEM. There was a significant trend for hematological improvement (*p < 0.05) in patients treated with Catvira when compared to the SOF + RBV treatment group.

Consistent with changes in laboratory values, Catvira treatment did not impact hemoglobin levels (Fig. 2a) or red blood cell (RBC) count (Fig. 2b), but the SOF + RBV individual tablets treatment resulted in a significant decline in both parameters after 24 weeks of treatment (*p* < 0.05).

**Figure 3:**
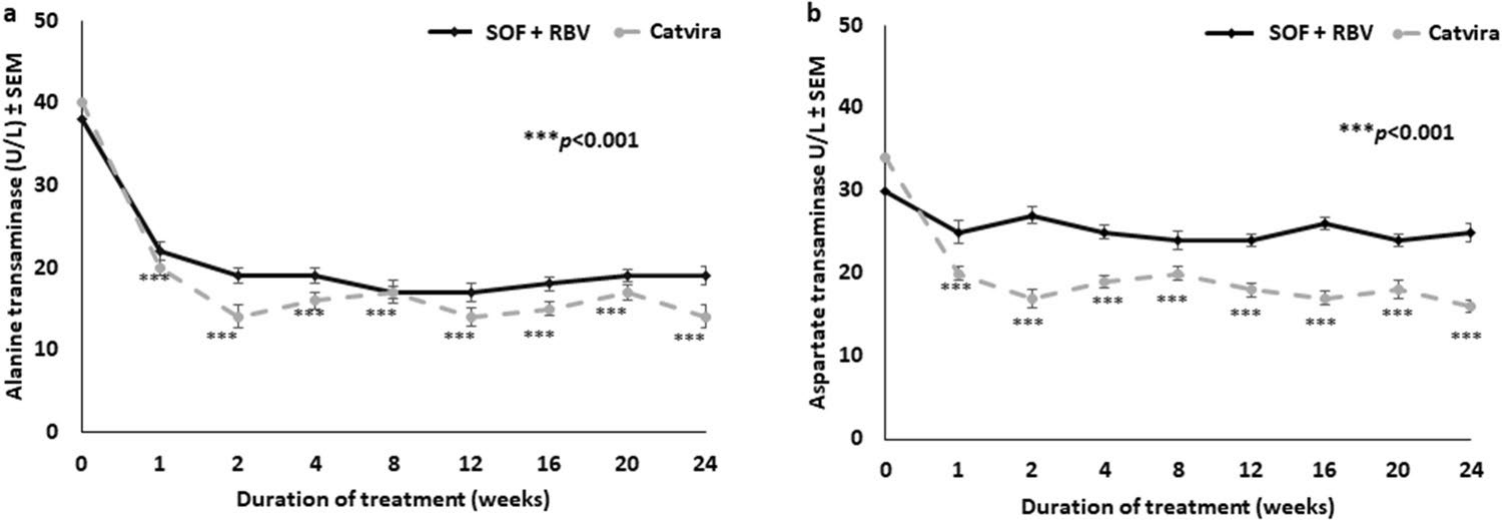
Effects on liver enzymes of Catvira treatment versus sofosbuvir (SOF) + ribavirin (RBV) individual tablets treatment in treatment-experienced patients with HCV (24 weeks treatment). (**a**) Effect on alanine transaminase (ALT) enzymes. (**b**) Effect on aspartate transaminase (AST) enzymes. Values are expressed as mean ± SEM. A significant difference (***p < 0.001) was observed in patients treated with Catvira and SOF + RBV when compared to control patients with HCV who were not treated.

We compared the effectiveness of Catvira treatment and the SOF + RBV individual tablets treatment on the liver functions by measuring ALT, AST, bilirubin, and alkaline phosphatase. Both treatments improved liver functions. Furthermore, both treatments achieved significant improvement (*p* < 0.001) in liver enzymes ALT (Fig. 3a) and AST (Fig. 3b).

There were no instances of a patient experiencing an adverse event that led to dose modification, interruption, or discontinuation of either regimen.

## Discussion

In this pilot study, 12 and 24 weeks of treatment with Catvira resulted in similar rates of SVR12 in treatment-naïve and treatment-experienced patients with genotype 4 HCV. The regimen was well tolerated, with mostly mild adverse events typically associated with RBV therapy, especially a decrease in RBC count and hemoglobin levels.

**Figure 4:**
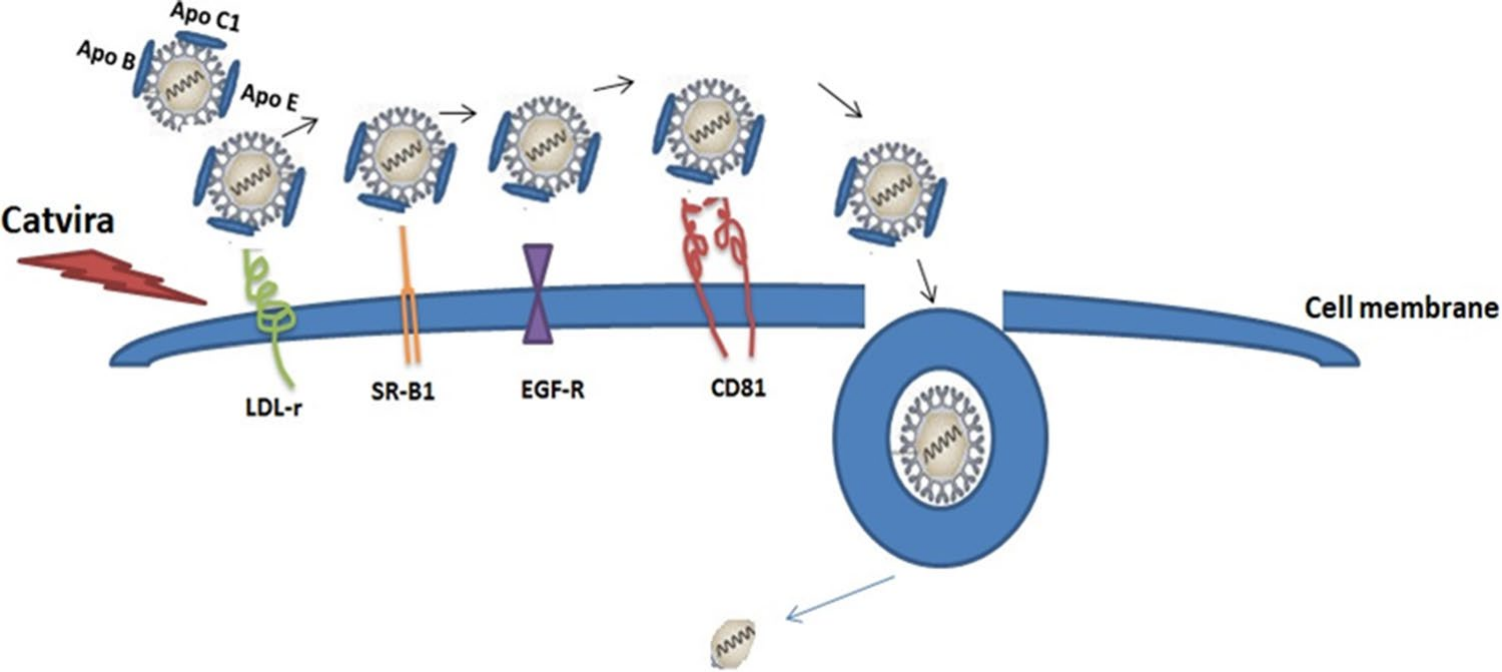
Catvira interferes with HCV entry into human hepatocytes. Cell entry involves an interaction between the extracellular virion that is associated with lipoproteins and several receptors on the host cell membrane. These include scavenger receptor type B class 1 (SR-B1), epidermal growth factor receptor (EGF-R), CD81, and possibly low-density lipoprotein receptor (LDL-r). It has been suggested that the lipoprotein receptors SR-B1 and LDL-r act before CD81. These interactions induce travelling of the virus-receptor complex along the cell surface from the basolateral (blood-side) surface of the hepatic epithelium where LDL-r, SR-B1, and CD81 are localised. These events, stimulated by virion-mediated activation of receptor tyrosine kinase signaling like EGF-R, result in clathrin-dependent endocytosis of the virion. Catvira is a single tablet composed of sofosbuvir (400 mg), ribavirin (1000 mg), and epigallocatechin gallate (EGCG) (400 mg). Catvira is suggested to act on the virus particle and inhibits virus entry by impairing virus binding to the cell surface.

Catvira consists of an additional molecule, EGCG, which is the bioactive catechin in green tea. EGCG displays strong preventive effects against different viral infections including HCV, HIV, influenza virus, and human herpes simplex virus^18^. Particularly, EGCG impairs the close links between the HCV life cycle and the lipid metabolism, which may interfere with HCV infection. Also, it has been reported that EGCG acts at an early step of entry by interfering with viral docking to the cell surface^19^. Initial binding of HCV particles to hepatocytes is mediated by heparin sulfate proteoglycan, scavenger receptor type B class 1 (SR-B1), and apoE^20–23^. Furthermore, the interaction of HCV particle with the low-density lipoprotein receptor (LDL-r) has also been proposed to play a role in the early phase of HCV entry^24^. Chen et al. reported that EGCG not only interferes with HCV entry, but also inhibits the viral RNA replication by 2- to threefold but only at a very high concentration (80 μM) of EGCG in vitro^25^. Further studies indicated that an observed bulge was found on the viral particle after EGCG treatment. This structural alteration led to a failure of membrane binding mediated by heparin sulfate- or sialic acid-containing glycans^14,26^. See Fig. 4 for an illustration of the potential effect of Catvira.

Lin et al. reported that HCV replication was downregulated by epicatechin isomers in vitro in cell-based HCV replicon and JFH-1 infectious systems. Epicatechin isomers have been reported to suppress the expression of inflammatory factors such as IL-1β, TNF-α, iNOS, and COX-2^27^.

EGCG is known to be safe with regard to hepatocytes. Calland et al. reported that EGCG had no toxic effect on Huh-7 cells (LD50 ~ 160 μM)^19^. Another study found that 400 mg of EGCG was well tolerated and safe in cirrhotic patients with HCV^28^.

In addition, EGCG has preventive effects against cardiovascular disease, metabolic syndrome, neurodegenerative diseases, and cancer^29^. EGCG might block hemolytic anemia, which is associated with RBV by acting as a highly efficient free radical scavenger. Consistent with our result, Kim et al. reported the protective effect of EGCG against the hemolytic effect of cyclosporine therapy, which was associated with increased production of free radical species^16^. Also, EGCG lowered the hemolysis and hydrogen peroxide levels and malondialdehyde produced by normal human erythrocytes (RBCs) incubated with cyclosporine.

Based on these preliminary data, Catvira might be superior to SOF + RBV individual tablet regimen with respect to adverse events, mainly RBV-induced hemolysis. Catvira not only interferes with viral replication, but also inhibits virus entry. However, any such conclusions need to be established with a larger number of patients.

Limitations of this study include the small sample size, no cirrhotic patients were included, and genotype subtypes were not done. Although most cases are now treated with an RBV-free regimen because of RBV’s side effects, RBV has a crucial role in increasing SVR for treatment-experienced and cirrhotic patients. Catvira is anti-hemolytic also; activity improves the tolerability of the therapy.

Catvira, administered daily for 12 or 24 weeks, is safe and effective in both naïve- and treatment-experienced patients with genotype 4 HCV. Catvira’s antiviral-entry mechanism may also play a role in enhancing efficacy and preventing relapse over SOF and RBV individual tablets. In addition to potentially enhanced efficacy, Catvira’s anti-hemolytic activity may improve the safety and tolerability of the therapy.

## Data Availability

The datasets generated during and/or analysed during the current study are available from the corresponding author on reasonable request.
